# From P_II_ Signaling to Metabolite Sensing: A Novel 2-Oxoglutarate Sensor That Details P_II_ - NAGK Complex Formation

**DOI:** 10.1371/journal.pone.0083181

**Published:** 2013-12-12

**Authors:** Jan Lüddecke, Karl Forchhammer

**Affiliations:** Interfaculty Institute for Microbiology and Infection Medicine (IMIT), Eberhard Karls University, Tübingen, Germany; University of Oldenburg, Germany

## Abstract

The widespread P_II_ signal transduction proteins are known for integrating signals of nitrogen and energy supply and regulating cellular behavior by interacting with a multitude of target proteins. The P_II_ protein of the cyanobacterium *Synechococcus elongatus* forms complexes with the controlling enzyme of arginine synthesis, N-acetyl-L-glutamate kinase (NAGK) in a 2-oxoglutarate- and ATP/ADP-dependent manner. Fusing NAGK and P_II_ proteins to either CFP or YFP yielded a FRET sensor that specifically responded to 2-oxoglutarate. The impact of the fluorescent tags on P_II_ and NAGK was evaluated by enzyme assays, surface plasmon resonance spectroscopy and isothermal calorimetric experiments. The developed FRET sensor provides real-time data on P_II_ - NAGK interaction and its modulation by the effector molecules ATP, ADP and 2-oxoglutarate *in vitro*. Additionally to its utility to monitor 2-oxoglutarate levels, the FRET assay provided novel insights into P_II_ - NAGK complex formation: (i) It revealed the formation of an encounter-complex between P_II_ and NAGK, which holds the proteins in proximity even in the presence of inhibitors of complex formation; (ii) It revealed that the P_II_ T-loop residue Ser49 is neither essential for complex formation with NAGK nor for activation of the enzyme but necessary to form a stable complex and efficiently relieve NAGK from arginine inhibition; (iii) It showed that arginine stabilizes the NAGK hexamer and stimulates P_II_ - NAGK interaction.

## Introduction

P_II_ proteins represent one of the most widespread and most ancient signal transduction protein families in nature. They are found in bacteria, archea and in chloroplasts of green algae and plants. P_II_ proteins act as signal processing units by integrating the cellular energy and nitrogen supply through binding of the central metabolites 2-oxoglutarate (2-OG), ATP and ADP in an interdependent manner [1-4]. The conformational changes induced by binding of these molecules enable P_II_ proteins to interact in a regulated manner with a wide range of partners including enzymes, transcription factors and membrane proteins. As typical for bacterial P_II_ proteins, the *Synechococcus elongatus* P_II_ protein forms homotrimers of 12.4 kDa subunits. At the bottom side of the cylindrically shaped trimer, a large and flexible loop (T‑loop), which takes part in protein interactions, protrudes from each subunit. The three effector binding sites are positioned in the three inter-subunit clefts, contacting the proximal part of the respective T‑loop, where ATP and ADP compete for binding, with a higher affinity for ATP [5]. Ligation of Mg^2+^ ATP further creates a binding site for 2-OG through the bridging Mg^2+^ ion. The three binding sites exhibit negative cooperativity towards each other, enabling P_II_ to sense the effector molecules in a wide range of concentrations [6-8]. Occupation of the ligand binding sites affects the conformation of the T‑loop, thereby regulating protein interactions.

In this paper we use the interaction between *S. elongatus* P_II_ and its partner N-acetyl-L-glutamate kinase (NAGK) to create a novel intermolecular FRET sensor able to read out the level of the central metabolite 2-OG. The P_II_ - NAGK complex formation has been thoroughly studied and is a model system to investigate fundamental aspects of PII-receptor interaction and signal transduction [3,5,7,9-11]. NAGK is a homohexameric enzyme (trimer of dimers) with a subunit size of 32.3 kDa. It catalyzes the committed step of the arginine synthesis pathway and is feed-back inhibited by arginine. If sufficient energy and nitrogen supplies are available, represented by high ATP and low 2-OG levels, two P_II_ trimers sandwich one NAGK hexamer [3]. The crystal structure of the P_II_ - NAGK complex revealed that each P_II_ subunit contacts a NAGK subunit at two sites: via its T‑loop, which folds in a unique conformation and by lateral contacts involving B-loop-residue Glu85. Based on mutational analysis, we hypothesized that a preliminary B-loop contact is the first step in complex formation, followed by structural rearrangement of the T‑loop [7]. The T-loop – NAGK interactions were regarded as decisive for the effects of P_II_ on the catalytic properties of NAGK: PII-binding boosts the activity of NAGK and relieves it from arginine feedback inhibition. If the cellular nitrogen supply is low and, as a result, 2-OG concentrations are high, P_II_ instead binds the effector molecules Mg^2+^ ATP and 2-OG, which results in a tightly folded T‑loop conformation that is unable to interact with NAGK [6,7,10]. The detailed molecular knowledge of the P_II_ - NAGK interaction motivated us to employ this system as a FRET-based biosensor for P_II_ effector molecules.

Resonance energy transfer was discovered by Förster in 1948 and is a non-radiative way of energy transfer between two dipoles. If used with fluorescent proteins (FPs) with spectral overlap between the donor emission and acceptor excitation the excited donor chromophor transfers its energy to the acceptor chromophor. This results in a decrease of donor fluorescence and an increase in acceptor fluorescence. The energy transfer works only on very small distances, generally less than 10 nm, and the efficiency of the transfer is directly related to the distance of the donor and acceptor proteins and also to their angle towards each other [12,13]. This makes FRET an ideal way to measure dynamic protein‑protein interaction *in vitro* and *in vivo* in real time and gives the system an advantage over other quantitative methods like surface plasmon resonance spectroscopy, which is not able to detect weak or slow interactions and needs one of the interaction partners to be bound on the detection chip. Although it has been shown that the addition of bulky FPs can alter the interaction of the tagged proteins [14], in most cases changes in biological activity were not reported or not investigated. Here we present the development of a FRET sensor for 2-OG, based on the interaction of P_II_ and NAGK and show that this FRET assay leads to novel insights into P_II_ - NAGK complex formation, not accessible to previous interaction assays. 

## Results and Discussion

### Generation of fusion protein constructs and influence of linkers on the FRET efficiency

The first effective FRET‑FP pair was developed by employing the GFP variants CFP and YFP as donor and acceptor fluorophores. It is still the most used FRET pair due to the great overlap of the CFP emission and the YFP excitation spectrum [15]. Here we use the third generation version of these proteins, Cerulean (derived from CFP) and Venus (derived from YFP). Cerulean shows improved fluorescence lifetime characteristics, a better quantum yield and a higher extinction coefficient. This leads to a 2.5 times improved fluorescence intensity compared to CFP [16]. Venus on the other hand is more resistant to low pH and chloride than YFP [17] and both proteins show enhanced maturation rates at 37 °C. To date the combination of Cerulean and Venus is one of the best general purpose FRET combinations and suitable for many different applications.

**Figure 1:**
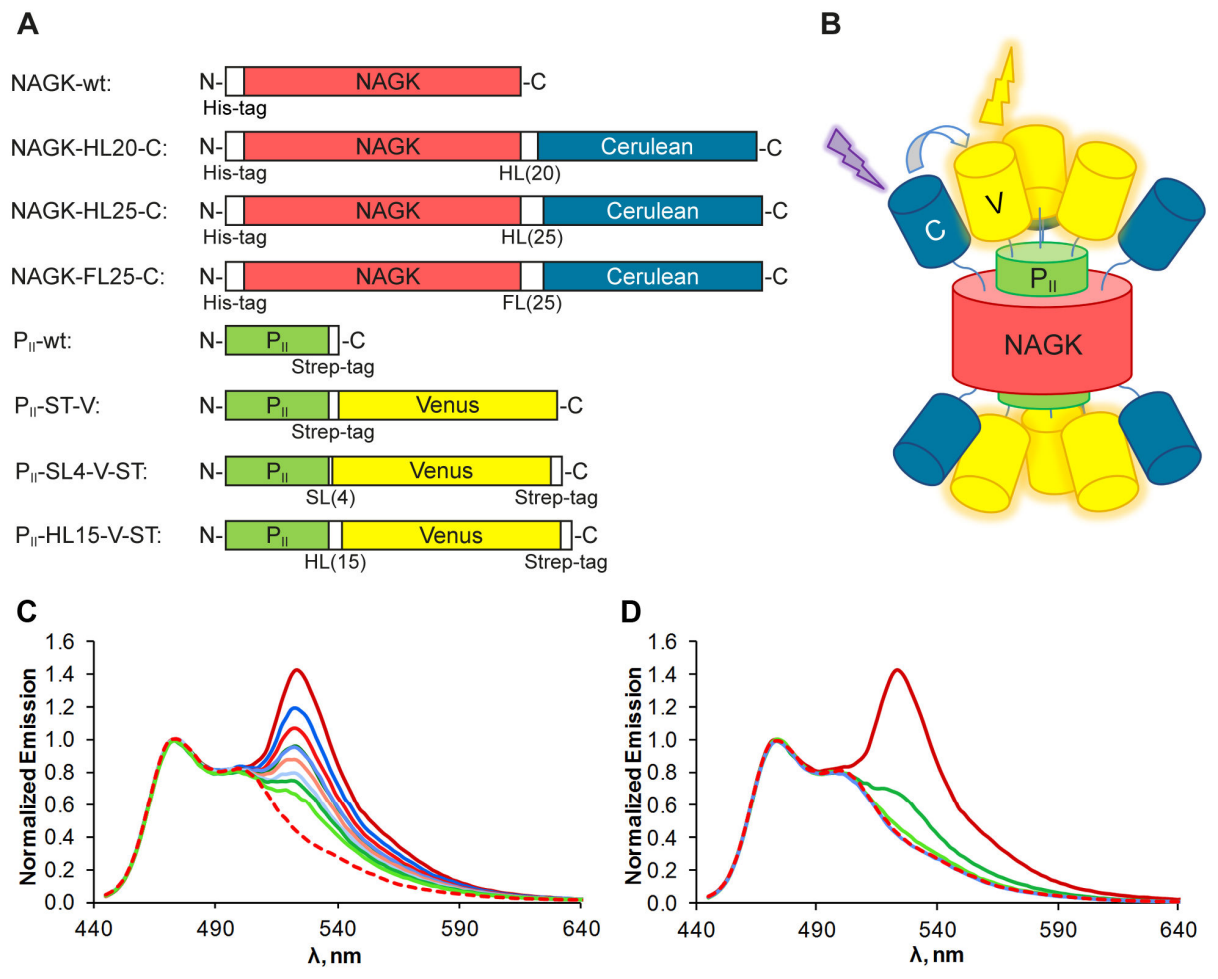
Fusion protein constructs and their FRET-performance. (**A**) Schematic representation of NAGK and P_II_ fusion proteins. Linker domains are denoted “HL” for helical linker, “FL” for flexible linker and “SL” for short linker with the corresponding number of amino acids in brackets. All domains are shown in the same scale. (**B**) Schematic representation of the assembled complex of FP-tagged P_II_ and NAGK. Two P_II_ trimers sandwich one NAGK hexamer. Cerulean domains are shown in blue, Venus domains are shown in yellow. (**C**) Emission spectra from different combinations of NAGK‑Cerulean and P_II_-Venus variants and NAGK‑FL25‑C alone, excited at 433 nm, emission scan from 445 to 640 nm. NAGK‑FL25‑C + P_II_-ST‑V in dark red, + P_II_-SL4‑V‑ST in red, + P_II_-HL15‑V‑ST in light red. NAGK‑HL20‑C + P_II_-ST‑V in dark blue, + P_II_-SL4‑V‑ST in blue, + P_II_-HL15‑V‑ST in light blue. NAGK‑HL25‑C + P_II_-ST‑V in dark green, + P_II_-SL4‑V‑ST in green, + P_II_-HL15‑V‑ST in light green and NAGK‑FL25‑C without P_II_ in dashed red. All spectra were corrected from background Venus emission and normalized to the Cerulean peak at 475 nm for better comparability. The peaks at 525 nm represent the Venus fluorescence induced by energy transfer from Cerulean. (**D**) Emission spectra from NAGK‑FL25‑C + P_II_-ST‑V in dark red, + P_II_-S49G‑ST‑V in green, + P_II_-E85A‑ST‑V in light green, + P_II_-S49G‑E85A‑ST‑V in blue and NAGK‑FL25‑C without P_II_ in dashed red. Note that the corrected spectrum recorded with the double mutant P_II_-S49G‑E85A‑ST‑V is identical to the spectrum of NAGK‑FL25‑C in the absence of P_II_.

Over the past years several crystal structures of *S. elongatus* P_II_ with its effector molecules and also together with NAGK could be solved [3,6,7,18]. Based on the structure of the P_II_ - NAGK complex we chose the C‑termini to fuse the FPs because these termini are freely accessible and point in the same direction. Linkers of different length and configuration were introduced between the native C‑termini and the FPs, as shown in Figure 1A, for several reasons: the FPs should not disturb the folding of the proteins nor impair protein interaction. Furthermore the Cerulean and Venus proteins should reside in close proximity upon P_II_ - NAGK complex formation. Therefore, we used a short leucine alanine linker (LAAA), flexible serine‑glycine linkers (L[SGGGG]_n_SAAA), and stiff helical linkers (A[EAAAK]_n_A). The latter have shown to be able to efficiently separate protein domains [19]. The NAGK and P_II_ proteins fused to the FPs should form a large complex with a molecular mass of approx. 619 kDa upon interaction (Figure 1B). The fusion proteins were overexpressed in *E. coli* and purified via Ni-NTA or Streptactin II affinity chromatography in high quantity and quality.

To test the FRET efficiency and the influence of the linkers, the purified NAGK fusion proteins were incubated with equimolar amounts of P_II_ fusion proteins. The fluorescence spectra were measured by exciting Cerulean with 433 nm light. To correct for unspecific Venus emission without FRET, the emission spectrum of P_II_-Venus was recorded in the absence of NAGK‑Cerulean and the spectrum was subtracted from the recorded FRET emission spectra. As illustrated in Figure 1C, all combinations showed FRET signals, indicating that all proteins formed functional oligomers and P_II_ and NAGK were able to interact despite the addition of the FPs. The efficiency of the energy transfer however differed considerably between the different constructs and combinations. Of the NAGK-Cerulean fusions the flexible linker variant generally yielded the highest energy transfers (reddish graphs), whereas the use of the long helical linker resulted in the lowest energy transfer (greenish graphs). Regarding the P_II_-Venus fusions, the use of the Strep‑tag as linker yielded the best results whereas again the helical linker fusions did show the lowest energy transfer. Possibly the stiffness of the helical linkers resulted in unfavorable conformations or a larger distance between the FPs. Accordingly, the combination of NAGK‑HL25‑C + P_II_-HL15‑V‑ST (for nomenclature definitions see Figure 1A) showed the lowest FRET and the combination of NAGK‑FL25‑C + P_II_-ST‑V showed the highest energy transfer and was, therefore, used for all following experiments. After having identified the optimal linker combinations, fusions were also constructed with P_II_ variants, which have been shown previously to be impaired in NAGK interaction, namely the variants P_II_-S49G and P_II_-E85A [3]. The aim was to create a non interacting P_II_ - NAGK pair that could serve as a negative control to better assess the FRET response of true interacting proteins. As shown in Figure 1D, the P_II_-S49G‑ST‑V variant yielded to our surprise a substantial FRET signal even though much lower than the wild-type form. The emission spectrum recorded from the NAGK‑FL25‑C + P_II_-E85A‑ST‑V pair, however, showed only a very low level of FRET. To completely abolish complex formation between P_II_ and NAGK, a P_II_-S49G‑E85A‑ST‑V double mutant was created. This combination indeed resulted in an emission spectrum that lacks FRET and just shows the Cerulean peak, like the measurement of NAGK‑FL25‑C alone.

### Influence of FP-tags on NAGK activity and P_II_ - NAGK complex formation

To compare the interaction between P_II_-ST-V and NAGK-FL25-C fusion proteins with the interaction of wild-type proteins, we conducted the hitherto established assays for complex formation, namely surface plasmon resonance spectroscopy (SPR) interaction assays and determination of catalytic properties of NAGK in presence or absence of PII.

**Figure 2:**
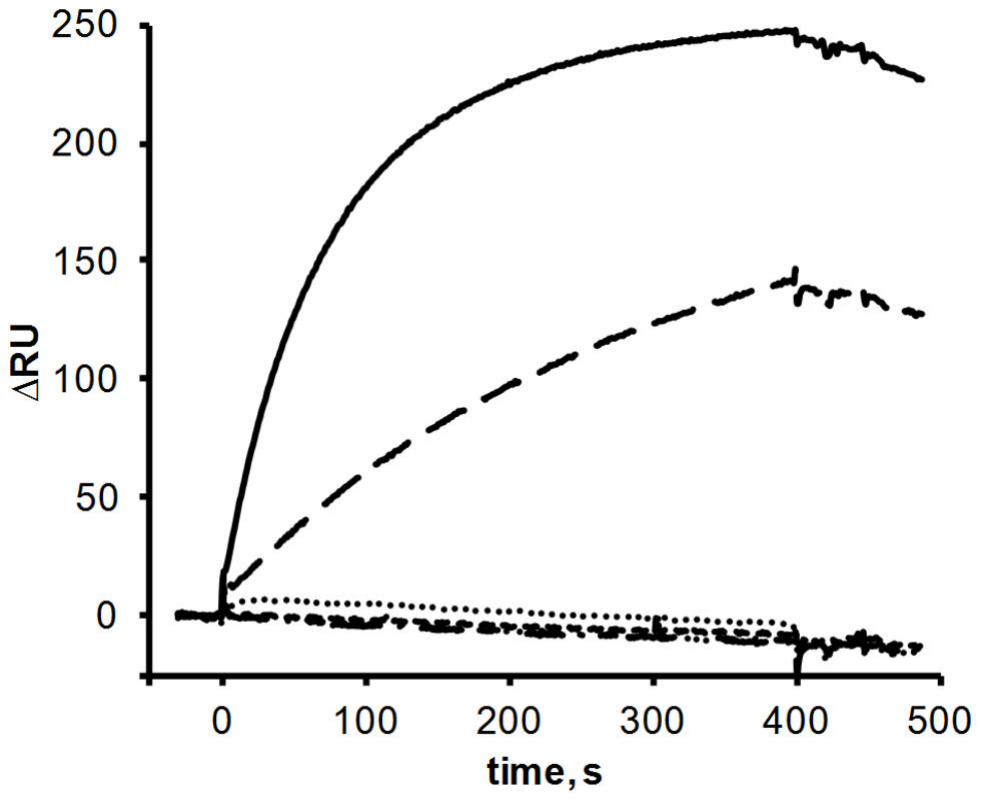
SPR analysis of the interaction of sensor-chip bound NAGK with P_II_ or Venus-tagged P_II_ variants. His-tagged NAGK protein was immobilized on the surface of flow cell (FC) 2 of a Ni-NTA coated sensor chip; FC 1 served as a control for unspecific binding. P_II_-wt (solid line), P_II_-ST‑V (long-dashed line), P_II_-S49G‑ST (dotted line), P_II_-S49G‑ST‑V (dashed line), P_II_-E85A-ST‑V (dot-dot-dashed line) and P_II_-S49G-E85A-ST‑V (dot-dashed line) were used as analyte. The response difference (∆RU) between FC2 and FC1 during injection and one minute of dissociation is shown.

First of all, we performed SPR experiments with NAGK‑FL25‑C bound to the sensor chip via its His-tag and using P_II_-ST‑V or P_II_-wt as analyte. Interestingly, no binding signal was measurable with any P_II_ variant, even at flow rates as low as 5 µl/min (data not shown), despite FRET experiments clearly showing interaction. These results indicate that either the interaction of P_II_ with Cerulean-fused NAGK is too slow to be detected by SPR or binding of NAGK‑FL25‑C with its His-tag to the chip surface shields the PII interaction site. By contrast, if the His-tagged NAGK-wt was bound to the sensor chip, interaction was measurable with P_II_-wt but also with the Venus-tagged P_II,_ although to a lower extent (Figure 2). A reason for this asymmetric dependence could be the different positions of the FP-tags: Cerulean is fused to NAGK near the P_II_ interaction site, whereas P_II_ is tagged with Venus on the far side of the interaction site. The Venus-tagged P_II_ variants S49G, E85A and the double mutant S49G‑E85A, but also the P_II_-S49G variant without Venus, did not bind to sensor-chip bound NAGK at all in the SPR experiments (Figure 2), like the P_II_-S49D variant tested before [3].

Next we studied the interaction of the FP-tagged P_II_ and NAGK proteins by enzyme assays. In wild-type proteins, the interaction of P_II_ with NAGK has two different consequences: first, NAGK is relieved from arginine inhibition and second, the enzymatic activity is greatly enhanced [20]. We determined the kinetic constants of NAGK-wt and NAGK‑FL25‑C alone and in combination with P_II_-wt and P_II_-ST‑V by using different concentrations of the substrate N-acetylglutamate (NAG). In the process of these experiments we noticed that it is beneficial for the NAGK activity to preincubate the enzyme in the reaction buffer. Only after a preincubation of at least 20 min at 25 °C NAGK reaches its maximum activity during the assay. Similar effects could be observed in FRET and SPR experiments. Possibly, NAGK needs time to reestablish its active hexameric conformation after storage in 50 % glycerol buffer at -20 °C. Due to this preincubation step, Kcat values were approximately 3 times higher and Km values 2 to 3 times lower compared to those published earlier [20]. 

The results in Table 1 show that the FPs have no negative influence on NAGK activity. In fact, the overall enzymatic activity (Kcat/Km) of NAGK‑FL25‑C is slightly higher than the activity of NAGK-wt (14 to 35 % increase). The activity boost induced by P_II_-ST‑V binding to NAGK is very similar to the one achieved by P_II_-wt. This demonstrates that the FPs do not affect the efficiency of complex formation. Contrary to our initial expectations, but confirmed by the FRET data (Figure 1B), the P_II_-S49G‑ST‑V variant was in fact able to activate NAGK almost in the same way as P_II_-wt does. 

**Table 1:** Kinetic constants and arginine inhibition properties (IC_50_) of NAGK-wt or NAGK‑FL25‑C in the absence or presence of various P_II_ variants.

NAGK	P_II_	Km (mM)	Kcat (s^-1^)	Kcat/Km (s^-1^ M^-1^)	IC_50_ Arg (µM)
NAGK-wt	-	3.2 ± 0.1	38.8 ± 0.5	1.2E+04	(20)
NAGK‑FL25‑C	-	3.0 ± 0.2	41.36 ± 0.5	1.4E+04	11 ± 1
NAGK-wt	P_II_-wt	0.7 ± 0.1	119.4 ± 1.6	1.8E+05	502 ± 20
NAGK‑FL25‑C	P_II_-wt	0.5 ± 0.0	128.5 ± 0.9	2.4E+05	n.d.
NAGK-wt	P_II_-ST‑V	0.7 ± 0.0	122.8 ± 1.0	1.8E+05	n.d.
NAGK‑FL25‑C	P_II_-ST‑V	0.6 ± 0.0	134.2 ± 1.0	2.1E+05	561 ± 32
NAGK-wt	P_II_49G‑ST‑V	0.5 ± 0.0	96.76 ± 1.3	1.9E+05	n.d.
NAGK‑FL25‑C	P_II_49G‑ST‑V	0.4 ± 0.0	99.61 ± 1.0	2.5E+05	170 ± 21

Original data and fitting to determine kinetic constants as well as IC_50_ Arg is shown in Figure S1. Km and Kcat values ± standard error. IC_50_ Arg ± 95% confidence interval. n.d., not determined.

As a second parameter for complex formation, the inhibition of NAGK activity by arginine and the relief of arginine inhibition by P_II_ addition were analyzed (Table 1). Arginine concentrations ranging from 1 µM to 1 mM were used in enzyme assays with NAGK-wt + P_II_-wt and NAGK‑FL25‑C + P_II_-ST‑V. Whereas the half maximal inhibitory concentration (IC_50_) of free NAGK-wt is approx. 20 µM [20], in presence of P_II_ the IC_50_ (± 95% confidence interval) increases 25 times to 502 (± 20) µM for the wt proteins, which is very similar to the values published before [7]. Free NAGK‑FL25‑C is also very sensitive to arginine with an IC_50_ of 11 (± 1) µM and P_II_-ST‑V is able to relieve arginine inhibition to an IC_50_ of 561(± 32) µM, similar to the effect observed for the wild-type proteins. Together these results show that the fluorescence-tags, despite their size, have only a minor impact on P_II_ - NAGK interaction. NAGK‑FL25‑C is as active as the wild-type enzyme, the activity is enhanced by P_II_ binding and P_II_ also relives NAGK from arginine inhibition. 

Comparing the SPR data with the results of enzyme analysis and FRET measurements reveals a striking difference: although P_II_-ST‑V seems to interact less strongly with NAGK than non-tagged P_II_ in SPR spectroscopy, no difference was recorded between tagged and non-fluorescence tagged proteins when measuring kinetic constants and relief from arginine inhibition, a sensitive indicator of complex formation. A further striking difference is the behavior of the P_II_-S49G variant: whereas the FRET measurements indicated the formation of a complex, no complex formation was seen by SPR, neither with the Venus-tagged form nor with the untagged variant. To resolve this issue, interaction of the P_II_-S49G variant with NAGK was also measured by the enzyme assay. The P_II_-S49G variant was indeed able to partially relief NAGK from arginine inhibition, raising the IC_50_ 8.5 times to 170 (± 21) µM. Thus P_II_-S49G is by far not as efficient in preventing arginine inhibition as P_II_-wt is, an intriguing difference to the almost wild-type like activation of NAGK in the absence of arginine. The differential effect of the P_II_-S49G mutation, on catalytic activation on one hand, and relief from arginine inhibition on the other, indicates that these two effects are transmitted by different intermolecular interactions. The comparison also shows that the different assays used report different aspects of complex formation. SPR is apparently very sensitive to steric hindrance caused by surface fixation of one interaction partner. Moreover, SPR fails to detect the interaction of the P_II_-S49G variant, indicating that the hydrogen bond of Ser49 is needed to stably fix P_II_ on surface-bound NAGK, although this variant is perfectly able to interact in solution with NAGK. On the other hand, efficient relief from arginine inhibition seems to require the formation of this hydrogen bond, whereas it seems dispensable for the basic activation of NAGK.

### ATP Binding to Venus-Tagged P_II_

To investigate whether the fusion of the FPs affects the effector molecule binding properties, isothermal titrations calorimetric (ITC) experiments were performed with P_II_-wt and P_II_-ST‑V proteins. The high binding affinity of P_II_-wt towards ATP has been shown before by equilibrium dialysis, ultrafiltration techniques [8] and ITC [7] and could be reproduced with dissociation constants of Kd1 = 4.8 ± 0.0 µM, Kd2 = 17.4 ± 1.5 µM and Kd3 = 92.8 ± 14.1 µM for the three binding sites with a sequential binding model (table 2). Interestingly, the affinity of P_II_-ST‑V towards ATP is doubled at the first binding site, compared to P_II_-wt, and is about 40 % higher at the second and third site.

**Table 2:** Determination of sequential binding of ATP to P_II_-wt or P_II_-ST‑V variant.

	Kd1 (µM)		Kd2 (µM)		Kd3 (µM)	
P_II_-wt	4.8	±0.0	17.4	±1.5	92.8	±14.1
P_II_-ST‑V	2.3	±0.5	10.9	±4.0	57.7	±3.3

Mean values of three replicas of the calculated ATP dissociation constants for the P_II_ binding sites 1-3 and ±SEM are shown. The original isotherms are presented in Figure S2.

Overall these analyses show that the fluorescence-tags have no inhibitory influence on effector molecule binding of P_II_. On the contrary, the tags lead to a higher affinity for ATP. As shown in the structure of liganded P_II_ [6], the C‑terminus of P_II_ flips towards the ATP biding site and shields it from the surface. The fused Venus protein may further constrict the ATP exit channel and therefore keep the bound ATP with higher affinity.

### P_II_ - NAGK binding kinetics and influence of effector molecules

After having characterized the principal properties of the FP tagged proteins and their mode of interaction, the FRET capabilities of the NAGK‑FL25‑C + P_II_-ST‑V pair was investigated in more detail. In the following experiments, Cerulean was excited with 433 nm light and the Cerulean and Venus fluorescence peak emission was recorded at 475 and 525 nm. The relative FRET ratio was calculated, which equals the emission at 525 nm (mainly Venus) divided by the emission at 475 nm (mainly Cerulean). First of all, we tested the impact of the ATP concentration on the protein interaction. NAGK‑FL25‑C and P_II_-ST‑V were incubated 30 min in the presence of different ATP concentrations and then FRET was measured. As shown in Figure S3, the strongest FRET signal is achieved with concentrations ranging from 0.1 to 1 mM ATP whereas 10 and 0.01 mM ATP result in a weaker signal. As ATP has no direct influence on the fluorescence of the tagged proteins (data not shown) this must be the result of an ATP induced conformational change of the P_II_ - NAGK interaction that has an influence on the energy transfer efficiency. In previously conducted SPR experiments, P_II_ and NAGK showed the same interaction with or without ATP if enough Mg^2+^ was present [10]. The FRET experiments, however, show a much weaker signal if no ATP is present. This leads to the conclusion, that without ATP P_II_ and NAGK still interact but are present in a less favorable conformation regarding FRET. In the following experiments we consequently used ATP concentrations of 0.1 mM.

**Figure 3:**
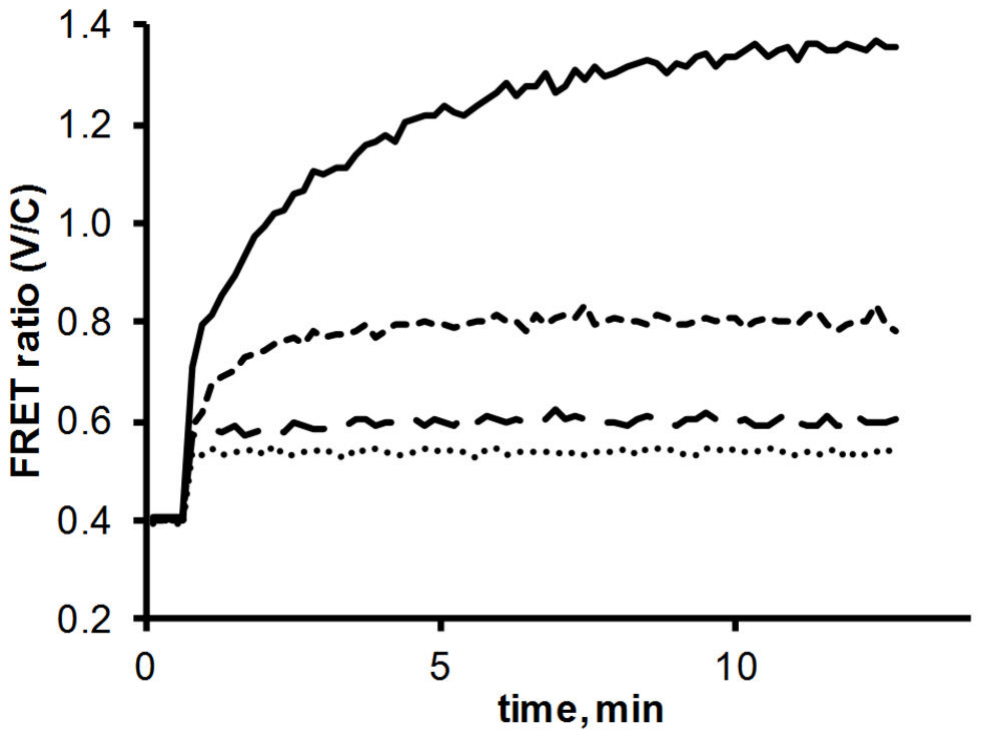
NAGK‑FL25‑C interaction with different P_II_-Venus-mutants: NAGK‑FL25‑C was preincubated in the reaction mix at 37 °C for 10 min. The measurement was started by recording the FRET ratio (525 nm / 475 nm emission) every 8 s. After two minutes the different P_II_ mutants were added: P_II_-ST‑V (solid line), P_II_-S49G‑ST‑V (dashed line), P_II_-E85A‑ST‑V (long-dashed line) and P_II_-S49G‑E85A‑ST‑V (dotted line).

Next we took a closer look at the association kinetics of FP-tagged NAGK, P_II_ and the P_II_ mutants. The FRET ratio was recorded every 8 s over 13 min as shown in Figure 3. In the absence of P_II_-ST‑V, the ratio is approx. 0.4, displaying pure Cerulean fluorescence. After recording the zero FRET response for 30 s, P_II_-ST‑V was added to the reaction. It immediately binds to NAGK‑FL25‑C, resulting in a fast increase in energy transfer. Following this initial raise the FRET ratio gradually saturates, reaching a maximum after 5 to 10 min.

The same experiment was also conducted with the P_II_ variants P_II_-S49G‑ST‑V, P_II_-E85A‑ST‑V and P_II_-S49G‑E85A‑ST‑V. The steady state FRET ratios are a lot lower in these experiments (Figure 3, dashed and dotted lines). The maximum FRET ratio of about 1.35 for P_II_-ST‑V compares with a ratio of 0.80 for P_II_-S49G‑ST‑V, 0.60 for P_II_-E85A‑ST‑V and 0.54 for the double mutant P_II_-S49G-E85A‑ST‑V. As shown before, the sudden increase from 0.4. to 0.54 after addition of the double mutant is not a result of FRET but represents the weak emission of Venus excited by 433 nm light. As such, this value represents the true zero baseline for the following experiments.

**Figure 4:**
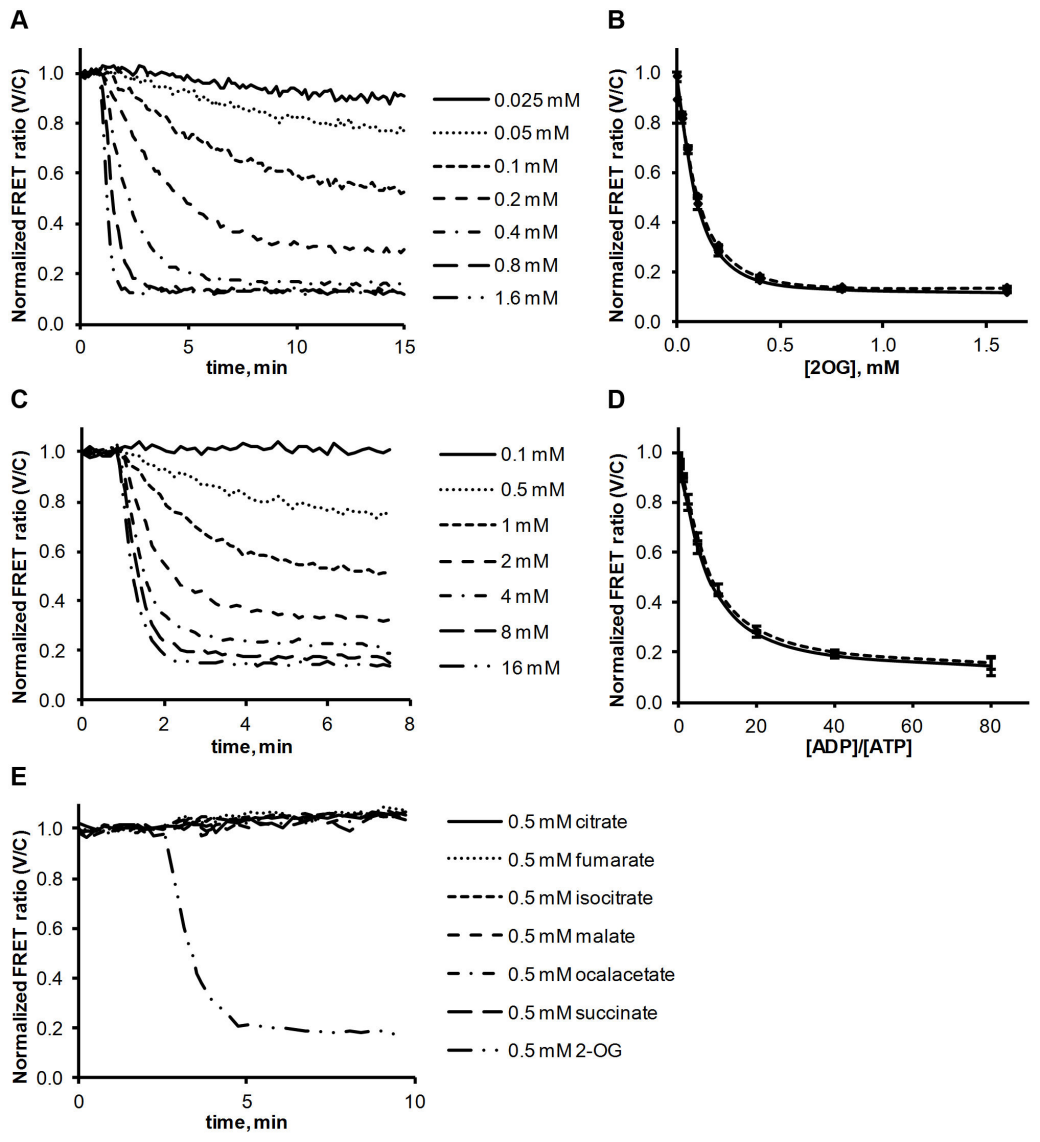
Influence of effector molecules on the P_II_ - NAGK complex. (**A**) 2-OG induced dissociation of the NAGK‑FL25‑C + P_II_-ST‑V complex, as determined by FRET analysis. The FRET ratio (525 nm / 475 nm emission) is background subtracted and normalized to first 6 values. (**B**) End point measurements of 2-OG induced dissociation. Solid line: NAGK‑FL25‑C and P_II_-ST‑V were incubated together for 10 min and then 2-OG was added. After 30 min of incubation the FRET ratio was determined. Dashed line: NAGK‑FL25‑C and P_II_-ST‑V were coincubated with 2-OG for 30 min without preincubation and then the FRET ratio was determined. All signals were normalized to values from control experiments without 2-OG. All reactions were performed as triplicates; standard deviation is indicated by error bars. (**C**) ADP-induced dissociation of the NAGK‑FL25‑C + P_II_-ST‑V complex, as determined by FRET analysis. Background subtracted and normalized to first 6 values. 0.1 mM ATP was present in the reaction mix. (**D**) End point measurements of ADP induced dissociation. Solid line: NAGK‑FL25‑C and P_II_-ST‑V were incubated together for 10 min and then ADP was added. After 30 min of incubation the FRET ratio was determined. Dashed line: NAGK‑FL25‑C and P_II_-ST‑V were coincubated with ADP for 30 min without preincubation and then the FRET ratio was determined. 0.1 mM ATP was present in the reaction mix, ADP concentrations were ranging from 0.25 to 8 mM. Inset: Magnification showing FRET signals from NAGK‑FL25‑C and P_II_-ST‑V in the presence of 2 mM ATP and ADP concentrations ranging from 0.5 to 2 mM (dotted line) representing more physiological concentrations. All signals were normalized to values from control experiments without ADP. All reactions were performed as triplicates; standard deviation is indicated by error bars. (**E**) Dissociation of NAGK‑FL25‑C and P_II_-ST‑V complex with 0.5 mM of malate, fumurate, succinate, oxalacetate, citrate, isocitrate and 2-OG. 0.075 mM ATP was present in all reactions.

The next step was to investigate the impact of different effector molecules on the P_II_ - NAGK FRET. First, NAGK and P_II_ were incubated together for 20 min to form the complex and then different concentrations of 2-OG or ADP were added. Figure 4A shows the baseline-subtracted FRET ratio, normalized to the initial values before addition of the effector molecules. Dissociation occurs in a concentration-depended manner, with higher concentrations of effectors leading to a faster FRET drop and resulting in lower steady-state levels.

Figure 4B shows end point measurements after 30 min of incubation with 2-OG. Two setups were used. In one experiment P_II_ and NAGK were pre-incubated for 10 min before 2-OG was added (solid line). In a parallel setup, 2-OG was already supplied to the reaction mix when P_II_ and NAGK were added and subsequently incubated for 30 min (dashed line). Both setups lead to the same endpoint FRET ratios, indicating that a preassembled P_II_ - NAGK complex responds towards 2-OG in the same manner as P_II_ before having bound to NAGK. The dynamic range for 2-OG detection is between 0.01 and 1.0 mM for the used conditions. The IC_50_, the 2-OG concentration that led to a 50 % drop in FRET, is 0.1 mM (Figure S4). These values correlates well with previous SPR and enzyme assays, indicating an IC_50_ of 0.12 mM [6]. Higher concentrations of 2-OG lead to a faster dissociation, but interestingly, the highest concentrations used, 0.8 and 1.6 mM, lead to the same steady state FRET level, which is 10 % above the background. This indicates a residual interaction of fully 2-OG complexed P_II_ with NAGK, which was not noticed previously. Since in the 2-OG liganded state the T-loop of P_II_ resides in a conformation incompatible with NAGK binding, this residual interaction could be mediated by the second interaction surface involving the B-loop of P_II_.

With a similar experimental setup as above, the response of the P_II_ - NAGK complex formation towards ADP was assayed, as shown in Figure 4C and D. As ATP and ADP compete for the same binding site, the dynamic range is of course strongly dependent on the ATP concentration present in the assay. In this case we used 0.1 mM ATP, since at this concentration optimal complex formation was recorded (see above). ADP concentrations ranged from 0.25 to 8 mM. Interestingly, very high ADP/ATP ratios of about 80 were necessary to achieve a maximal FRET drop, which again was 10 % above the baseline. The IC_50_ for the ADP/ATP ratio is at about 10, which means that the FRET drops to 50 % if 10 times more ADP then ATP is present (Figure S4). We also tested the influence of ADP under more physiological conditions, using 2 mM ATP and ADP concentrations ranging from 0.5 to 2 mM (Figure 4D inset). The results are very similar to those with lower ATP concentrations. This shows that not the absolute concentrations are important but the ratio of ADP and ATP. It also means that *in vivo* ADP has almost no effect on the P_II_ - NAGK interaction, as a 1:1 ADP:ATP ratio leads to a FRET loss of only 10 % and in *E. coli* for example ATP concentrations are generally 17 to 60 times higher than ADP concentrations under exponential growth conditions [21]. The results obtained correlate well with previous results showing that *S. elongatus* P_II_ shows a 2 - 3 times lower affinity for ADP than ATP [7] contrary to *E. coli* P_II_ proteins which have a higher affinity for ADP [22]. On the other hand, previous SPR experiments already showed one third loss of P_II_ - NAGK binding with 1:1 ATP/ADP ratios although this might be a result of the artificial conditions of the SPR experiment [5].

In the next experiments, the specificity of P_II_ for 2-OG was tested by adding different intermediates of the TCA cycle to the assembled P_II_ - NAGK complex. 0.5 mM concentrations of isocitrate, succinate, fumarate, malate, oxaloacetate, or citrate did not have any effect on the FRET signal (Figure 4E). 

**Figure 5:**
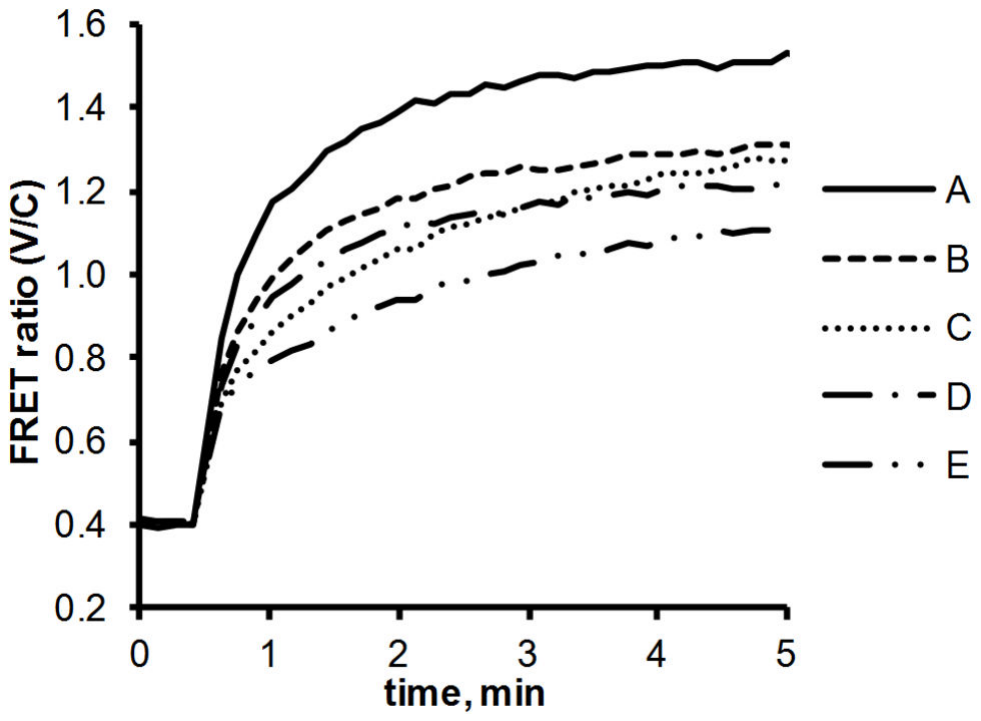
Influence of arginine and NAGK preincubation on P_II_ - NAGK complex formation. Before the measurement was started: A: NAGK‑FL25‑C was preincubated 40 min in the presence of 1 mM arginine. B: NAGK‑FL25‑C was preincubated 40 min and then arginine (1 mM) was added. C: NAGK‑FL25‑C was preincubated 40 min. No arginine was present. D: NAGK‑FL25‑C was preincubated 5 min and then arginine (1 mM) was added. E: NAGK‑FL25‑C was preincubated 5 min. No arginine was present. The measurement was started by recording the FRET ratio (525 nm / 475 nm emission) every 8 s. P_II_-ST‑V was added after 30 s.

Finally, the novel FRET assay was used to study the effect of arginine on P_II_ - NAGK complex formation. SPR analysis failed to reveal any inhibitory effect of arginine on P_II_ - NAGK complex formation [10]. However, since the SPR assay has its limitations (see above), we wanted to confirm this result by the FRET assay. Figure 5 shows FRET assembly kinetics of P_II_ and NAGK in the presence or absence of 1 mM arginine and with different NAGK preincubation times. Interestingly, these results show that arginine has a strong positive effect on the assembly of the P_II_ - NAGK complex, demonstrated by the fast raise of the FRET ratio, especially if NAGK is preincubated 40 min in the presence of arginine before P_II_ is added (Figure 5A). If arginine is added together with P_II_, the assembly is a lot slower (Figure 5B), but still faster than an assembly reaction completely without arginine (Figure 5C). In experiments D and E, the preincubation time of NAGK was lowered to 5 min. Here, the assembly is even slower, but the addition of arginine still has a pronounced positive effect (Figure 5D). From these results it can be concluded that for an efficient interaction between P_II_ and NAGK the stability of the NAGK hexamer is essential. Although a long preincubation time is beneficial for NAGK hexamerisation, the addition of arginine seems to be even more efficient to assemble the NAGK enzyme in a conformation that avidly binds P_II_. To investigate if the positive effect of arginine on the complex formation also alters the sensitivity towards 2-OG, we incubated P_II_ and NAGK together with different 2-OG concentrations and with or without 1 mM arginine. Indeed the addition of arginine has a stabilizing effect on the complex, which renders it less sensitive towards 2-OG induced dissociation, resulting in a 30 % increased IC_50_ for 2-OG (Figure S5).

These results also clearly demonstrate that the inhibitory effect of arginine on the enzymatic activity of the P_II_ - NAGK complex is not mediated by dissociation of the complex but by a rearrangement of NAGKs active site in the presence of bound P_II_.

Together, the characterization of P_II_ - NAGK interaction by the novel FRET system led to important new insights in the details of P_II_ - receptor complex formation: We postulated earlier that P_II_ encounters NAGK by contacts involving the B‑loop region and assembles tightly with NAGK only after a structural rearrangement of the T‑loop [7]. The present results perfectly agree with this model: Mutation of the crucial B‑loop amino acid Glu85 to Ala greatly reduces FRET efficiency of NAGK interaction by impairing formation of the postulated encounter complex. By contrast, exchange of Ser49 to Gly has a less severe effect on formation of the complex in solution. This indicates that a complex can be formed even in the absence of the hydrogen bond of Ser49, which was regarded previously as essential [3]. This hydrogen bond is apparently only required to stably fix P_II_ into the NAGK binding pocket, but is not needed to activate the catalytic capacity of NAGK, since the P_II_-S49G variant could perfectly activate NAGK. The residual FRET signal, which was observed when P_II_ was liganded either with 2-OG or ADP, indicates that the interaction of P_II_ with NAGK is not completely abolished under these conditions, but that a transient interaction still occurs. This transient interaction has no effect on the catalytic properties of NAGK as compared to the absence of P_II_, but would allow a rapid re-establishment of the fully functional P_II_ - NAGK complex, once the unfavorable effector has dissociated. On the other hand, arginine binding to the allosteric binding sites helps NAGK to adopt a conformation that favors P_II_ binding, so that P_II_ can alleviate the inhibitory effect of the feedback inhibitor.

## Conclusions

The interaction of *S. elongatus* P_II_ with NAGK was used to develop a biosensor able to measure 2-OG in sub mM concentrations. The study showed that P_II_, although being a homotrimer, is suitable for FRET applications. The possibility of using P_II_ for FRET approaches opens a wide field of possible sensor applications as P_II_ proteins have many different interaction partners whose interactions depend on different metabolic conditions. We assume that the P_II_ - NAGK sensor system should in principle also work for *in vivo* applications, where it could work as a pure 2-OG sensor because the ADP/ATP ratios of living cells do not affect the 2-OG mediated signal output. This sensor also led to new insights in details of the interaction between P_II_ and NAGK, which were not possible to analyze before. The NAGK hexamer needs to arrange in a quaternary structure that allows efficient and fast interaction with P_II_. Although arginine is an inhibitor of NAGKs enzymatic activity, it helps NAGK to adopt the P_II_-interacting conformation, presumably by binding to the allosteric sites near the interface between the subunits. For the first time, we could directly observe different stages of P_II_ - NAGK complex formation. FRET revealed an encounter complex that does not involve the T-loop of P_II_. Furthermore, a preliminary complex involving the T-loop was detected that is sufficient to activate the catalytic centre of NAGK but does not require the Ser49 mediated hydrogen bonds. Only after the hydrogen bonds of Ser49 are formed, a stable complex is established that results in maximal relief from arginine inhibition. This FRET sensor has, therefore, a great potential to elucidate in detail the various processes of P_II_-receptor interactions. 

## Materials and Methods

### Cloning, overexpression and purification of the fusion proteins

Cloning was conducted using standard PCR techniques. Oligonucleotides were synthesized by Eurofins MWG GmbH (Ebersberg, Ger) or Sigma Aldrich Chemie GmbH (Steinheim, Ger). Restriction enzymes were purchased from Fisher Scientific (Schwerte, Ger). PCR products and digested DNA were purified using QIAquick gel extraction kits (QIAGEN GmbH, Hilden, Ger). Plasmid DNA from *E. coli* Dh5α over night cultures was purified using peqGOLD plasmid miniprep kits (PEQLAB Biotechnologie GMBH, Erlangen, Ger). The vector pASK-IBA3 (IBA GmbH, Göttingen, Ger) with *tet*-promotor and a Strep‑tag coding sequence was used for P_II_ expression. The vector pET-15b (Merck KGaA, Darmstadt, Ger) with T7-promotor and a His-tag coding sequence was used for the expression of NAGK. DNA sequences for the GFP variants Venus and Cerulean were amplified from pSW41, a generous gift from Sabrina Wend, University of Freiburg, Freiburg, Germany. The linker sequences were generated by overlapping oligo PCRs. *E. coli* strain RB9060 [23] was transformed with the P_II_ plasmids and the proteins were purified using Strep‑tag affinity chromatography as described earlier [9]. *E. coli* BL21 cells were transformed with the NAGK plasmids and the proteins were purified using His-tag affinity chromatography as previously described [10].

### FRET measurements

Fluorescence measurements were performed using a FluoroMax‑2 (HORIBA Europe GmbH, Berlin, Ger). If not otherwise described the reaction buffer contained 50 mM imidazole (pH 7.5), 50 mM KCl, 20 mM MgCl_2_, 0.5 mM DTT and 0.1 mM ATP. The fusion proteins were used in concentrations of 100 nM. Prior measurements NAGK was carefully transferred into the reaction buffer and incubated for 10 min on ice and after that 20 to 40 min at 37 °C. To measure association kinetics P_II_-Venus fusions were added, the mixture was excited by 433 nm and Cerulean and Venus emission peaks were measured at 475 and 525 nm wavelengths every 8 s. Between the measurements the shutter was closed to prevent bleaching. Fluorescence spectra were measured using an emission scan ranging from 445 to 650 nm with excitation at 433 nm wavelength. The bandpass for emission and excitation was set to 1.5 nm in all experiments.

### Assay of NAGK activity by coupling NAG phosphorylation to NADH oxidation

The NAGK activity assay was performed in similar ways as described before regarding buffers and the measurement setup [20,24]. But to yield maximum activity of NAGK the enzyme was preincubated in a buffer containing 50 mM imidazole, pH 7.5, 50 mM KCl, 20 mM MgCl2, 0.5 mM DTT, and 0.1 mM ATP at 25 °C for 20 to 40 min. If NAGK activity was to be measured in the presence of P_II_, then both proteins were preincubated together. 50 µl of the preincubation mix were added to the reaction mix resulting in final concentrations of 50 mM imidazole, pH 7.5, 50 mM KCl, 20 mM MgCl2, 0.4 mM NADH, 1 mM phosphoenolpyruvate, 10 mM ATP, 0.5 mM DTT, 11 U lactate dehydrogenase, 15 U pyruvate kinase and 25 nM of NAGK and P_II_. The reaction was started by adding varying concentrations of the substrate NAG, resulting in a final reaction volume of 1 ml, and the NADH oxidation was immediately assessed by measuring the absorbance at 340 nm with a SPECORD 200 photometer (Analytik Jena) for 60 s. The slope of the change of absorbance over time was calculated. One unit of NAGK catalyses the conversion of 1 µmol NAG min^−1^, calculated with a molar absorption coefficient of NADH of ɛ340 = 6178 L mol^−1^ cm^−1^. GaphPad Prism 6 (GraphPad Software, San Diego) was used to calculate the enzymatic parameters Km, Kcat, and IC_50_.

### Surface plasmon resonance experiments

SPR experiments were performed using a using a BIAcore X biosensor system (Biacore) as previously described [10]. His-tagged NAGK (30 nM hexamer) was bound to the Ni^2+^ loaded NTA surface of flow cell (FC) 1 until a signal of ~1500 resonance units was reached. FC 2 acted as a control for unspecific binding and the signal was subtracted from FC 1. Variants of P_II_ were injected to the sensor chip and, after binding, removed again by injecting a 1 mM ADP solution.

### Isothermal titration calorimetry

ITC experiments were performed using a VP‑ITC microcalorimeter (MicroCal) in buffer containing 10 mM Na_2_HPO_4_, 1.8 mM KH_2_PO_4_, 25 mM NaCl, 10 mM KCl and 1 mM MgCl_2_ (pH 7.5). 1 mM of the titrant ATP was dissolved in the same buffer. After an initial 2 µl injection 4 µL of the titrant were injected 70 times into the 1.4285 ml cell with stirring at 200 rpm at a temperature of 25 °C. The P_II_ concentration in the cell was 33 µM (trimer concentration). The binding isotherms were calculated from received data and fitted to a three-site binding model with the MicroCal ORIGIN software (Northampton, MA) as indicated.

## Supporting Information

Figure S1 **Determination of kinetic constants for NAGK-wt and NAGK‑FL25-C with and without various P_II_ variants.** (A) NAGK enzyme activity assays of the indicated P_II_ - NAGK combinations were performed using increasing amounts of the substrate NAG. (B) NAGK enzyme activity assays of the indicated P_II_ - NAGK combinations were performed with increasing concentrations of Arginine and 50 mM NAG. The mean of three replicas with error bars displaying the standard deviation is shown. The results are summarized in Tab. 1.
(TIF)

Figure S2 **Two exemplary Isotherms for the determination of ATP binding properties of P_II_-wt and P_II_-ST‑V.** The upper panel shows the raw data, the lower panel shows the calculated, blank subtracted, binding isotherms. The results are summarized in Table 2.
(TIF)

Figure S3 **Effect of various ATP concentrations on FRET efficiency of NAGK‑FL25‑C with P_II_-ST‑V.** The FRET ratio (525 nm / 475 nm emission) was measured after incubating NAGK-FL25-C and P_II_-ST-V together for 20 min at 37 °C in the presence of different ATP concentration. The mean of three replicas with error bars displaying the standard deviation is shown.
(TIF)

Figure S4 **Determination of IC_50_ values for 2-OG and ADP on NAGK‑FL25‑C + P_II_-ST‑V.** The relative FRET ratio (525 nm / 475 nm emission) was measured after incubating NAGK-FL25-C and P_II_-ST-V together for 30 min in the presence of different 2-OG concentration (A) or ADP/ATP ratios (B). All signals were normalized to values from control experiments without 2 OG or ADP. The mean of three replicas with error bars displaying the standard deviation is shown. Fitting and IC_50_ calculation was conducted using GaphPad Prism 6.
(TIF)

Figure S5 **Influence of arginine on P_II_ - NAGK complex sensitivity towards 2-OG.** P_II_-ST-V and NAGK-FL25-C were incubated together for 40 min at 37 °C with different concentrations of 2-OG and with or without 1 mM arginine, before the FRET ratio was determined. All reactions were performed as triplicates; standard deviation is indicated by error bars.
(TIF)

## References

[1] Forchhammer K (2008) P(II) signal transducers: novel functional and structural insights. Trends Microbiol 16: 65-72. doi:10.1016/j.tim.2007.11.004. PubMed: 18182294. 18182294

[2] Leigh JA, Dodsworth JA (2007) Nitrogen regulation in bacteria and archaea. Annu Rev Microbiol 61: 349-377. doi:10.1146/annurev.micro.61.080706.093409. PubMed: 17506680. 17506680

[3] Llácer JL, Contreras A, Forchhammer K, Marco-Marín C, Gil-Ortiz F et al. (2007) The crystal structure of the complex of PII and acetylglutamate kinase reveals how PII controls the storage of nitrogen as arginine. Proc Natl Acad Sci U S A 104: 17644-17649. doi:10.1073/pnas.0705987104. PubMed: 17959776. 17959776 PMC2077032

[4] Ninfa AJ, Jiang P (2005) PII signal transduction proteins: sensors of alpha-ketoglutarate that regulate nitrogen metabolism. Curr Opin Microbiol 8: 168-173. doi:10.1016/j.mib.2005.02.011. PubMed: 15802248. 15802248

[5] Fokina O, Herrmann C, Forchhammer K (2011) Signal-transduction protein PII from Synechococcus elongatus PCC 7942 senses low adenylate energy charge in vitro. Biochem J 440: 147-156. doi:10.1042/BJ20110536. PubMed: 21774788. 21774788

[6] Fokina O, Chellamuthu V-R, Forchhammer K, Zeth K (2010) Mechanism of 2-oxoglutarate signaling by the Synechococcus elongatus PII signal transduction protein. Proceedings of the National Academy of Sciences of the USA 107: 19760-19765. doi:10.1073/pnas.1007653107. PubMed: 21041661. 21041661 PMC2993416

[7] Fokina O, Chellamuthu VR, Zeth K, Forchhammer K (2010) A novel signal transduction protein P(II) variant from Synechococcus elongatus PCC 7942 indicates a two-step process for NAGK-P(II) complex formation. J Mol Biol 399: 410-421. doi:10.1016/j.jmb.2010.04.018. PubMed: 20399792. 20399792

[8] Forchhammer K, Hedler A (1997) Phosphoprotein PII from cyanobacteria--analysis of functional conservation with the PII signal-transduction protein from Escherichia coli. Eur J Biochem 244: 869-875. doi:10.1111/j.1432-1033.1997.00869.x. PubMed: 9108259. 9108259

[9] Heinrich A, Maheswaran M, Ruppert U, Forchhammer K (2004) The Synechococcus elongatus P signal transduction protein controls arginine synthesis by complex formation with N-acetyl-L-glutamate kinase. Mol Microbiol 52: 1303-1314. doi:10.1111/j.1365-2958.2004.04058.x. PubMed: 15165234. 15165234

[10] Maheswaran M, Urbanke C, Forchhammer K (2004) Complex formation and catalytic activation by the PII signaling protein of N-acetyl-L-glutamate kinase from Synechococcus elongatus strain PCC 7942. J Biol Chem 279: 55202-55210. doi:10.1074/jbc.M410971200. PubMed: 15502156. 15502156

[11] Zeth K, Fokina O, Forchhammer K (2012) An engineered PII protein variant that senses a novel ligand: atomic resolution structure of the complex with citrate. Acta Crystallogr D Biol Crystallogr 68: 901-908. doi:10.1107/S0907444912016447. PubMed: 22868755. 22868755

[12] Stryer L, Haugland RP (1967) Energy transfer: a spectroscopic ruler. Proc Natl Acad Sci U S A 58: 719-726. doi:10.1073/pnas.58.2.719. PubMed: 5233469. 5233469 PMC335693

[13] Förster T (1948) Intermolecular Energy Migration and Fluorescence. Ann Phys 2: 55–75.

[14] Hoffmann C, Gaietta G, Bünemann M, Adams SR, Oberdorff-Maass S et al. (2005) A FlAsH-based FRET approach to determine G protein-coupled receptor activation in living cells. Nat Methods 2: 171-176. doi:10.1038/nmeth742. PubMed: 15782185. 15782185

[15] Miyawaki A, Llopis J, Heim R, McCaffery JM, Adams JA et al. (1997) Fluorescent indicators for Ca2+based on green fluorescent proteins and calmodulin. Nature 388: 882-887. doi:10.1038/42264. PubMed: 9278050. 9278050

[16] Rizzo MA, Springer GH, Granada B, Piston DW (2004) An improved cyan fluorescent protein variant useful for FRET. Nat Biotechnol 22: 445-449. doi:10.1038/nbt945. PubMed: 14990965. 14990965

[17] Nagai T, Ibata K, Park ES, Kubota M, Mikoshiba K et al. (2002) A variant of yellow fluorescent protein with fast and efficient maturation for cell-biological applications. Nat Biotechnol 20: 87-90. doi:10.1038/nbt0102-87. PubMed: 11753368. 11753368

[18] Xu Y, Carr PD, Clancy P, Garcia-Dominguez M, Forchhammer K et al. (2003) The structures of the PII proteins from the cyanobacteria Synechococcus sp. PCC 7942 and Synechocystis sp. PCC 6803. Acta Crystallogr D Biol Crystallogr 59: 2183-2190. PubMed: 14646076. 14646076 10.1107/s0907444903019589

[19] Arai R, Ueda H, Kitayama A, Kamiya N, Nagamune T (2001) Design of the linkers which effectively separate domains of a bifunctional fusion protein. Protein Eng 14: 529-532. doi:10.1093/protein/14.8.529. PubMed: 11579220. 11579220

[20] Beez S, Fokina O, Herrmann C, Forchhammer K (2009) N-Acetyl-l-Glutamate Kinase (NAGK) from Oxygenic Phototrophs: PII Signal Transduction across Domains of Life Reveals Novel Insights in NAGK Control. J Mol Biol 389: 748-758. doi:10.1016/j.jmb.2009.04.053. PubMed: 19409905. 19409905

[21] Bennett BD, Kimball EH, Gao M, Osterhout R, Van Dien SJ et al. (2009) Absolute metabolite concentrations and implied enzyme active site occupancy in Escherichia coli. Nat Chem Biol 5: 593-599. doi:10.1038/nchembio.186. PubMed: 19561621. 19561621 PMC2754216

[22] Jiang P, Ninfa AJ (2007) Escherichia coli PII Signal Transduction Protein Controlling Nitrogen Assimilation Acts As a Sensor of Adenylate Energy Charge in Vitro†. Biochemistry 46: 12979-12996. doi:10.1021/bi701062t. PubMed: 17939683. 17939683

[23] Bueno R, Pahel G, Magasanik B (1985) Role of glnB and glnD gene products in regulation of the glnALG operon of Escherichia coli. J Bacteriol 164: 816-822. PubMed: 2865248. 2865248 10.1128/jb.164.2.816-822.1985 PMC214324

[24] Jiang P, Ninfa AJ (1999) Regulation of Autophosphorylation of Escherichia coli Nitrogen Regulator II by the PII Signal Transduction Protein. J Bacteriol 181: 1906-1911. PubMed: 10074086. 10074086 10.1128/jb.181.6.1906-1911.1999 PMC93592

