# Correction: From P_II_ Signaling to Metabolite Sensing: A Novel 2-Oxoglutarate Sensor That Details P_II_ - NAGK Complex Formation

**DOI:** 10.1371/journal.pone.0103759

**Published:** 2014-07-22

**Authors:** The PLOS ONE Staff

Figure 4 is incorrect. The authors have provided a corrected Figure 4 here.

**Figure 4:**
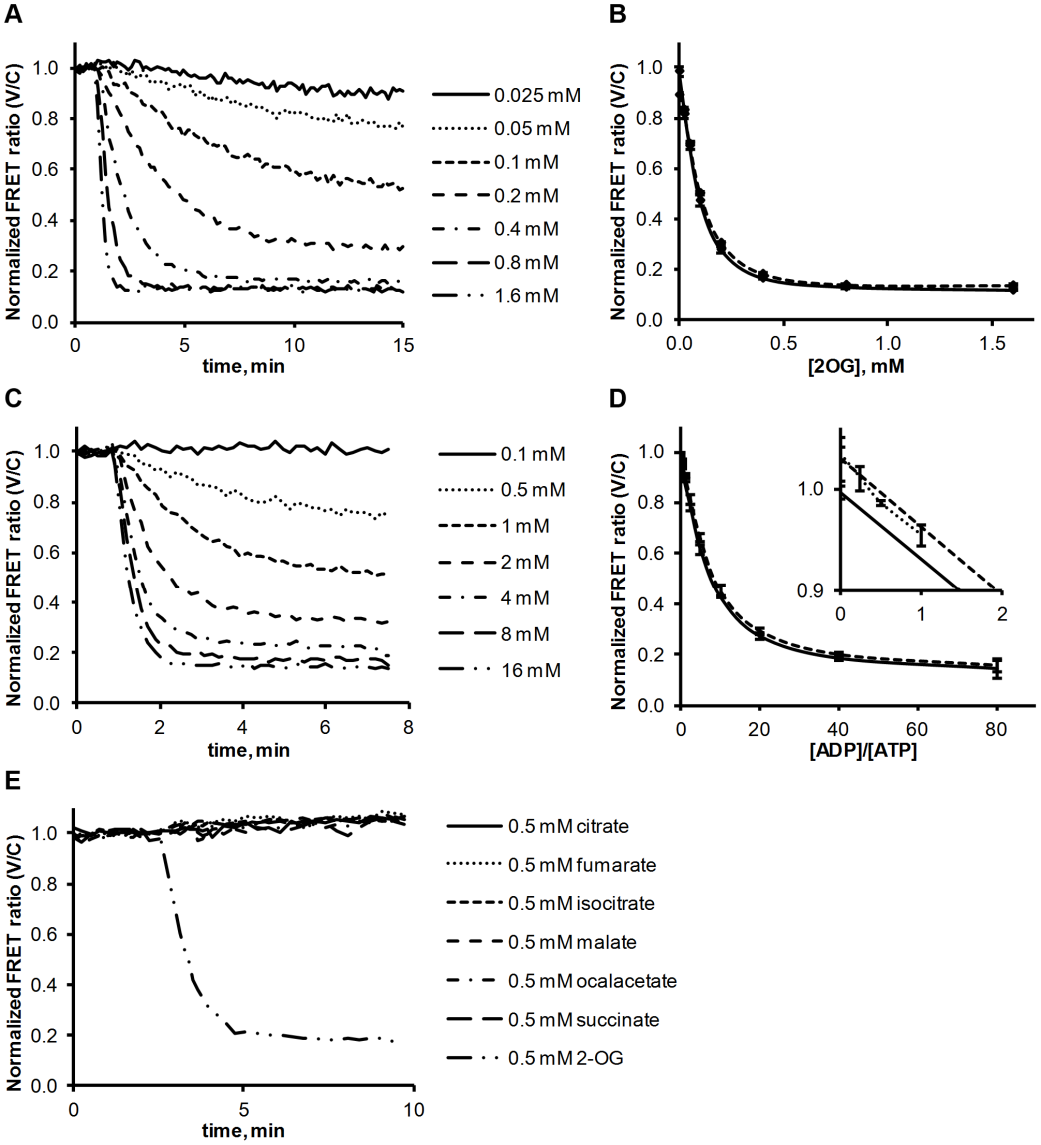
Influence of effector molecules on the P _II_ - NAGK complex. (A) 2-OG induced dissociation of the NAGK-FL25-C + PII-ST-V complex, as determined by FRET analysis. The FRET ratio (525 nm / 475 nm emission) is background subtracted and normalized to first 6 values. (B) End point measurements of 2-OG induced dissociation. Solid line: NAGK-FL25-C and PII-ST-V were incubated together for 10 min and then 2-OG was added. After 30 min of incubation the FRET ratio was determined. Dashed line: NAGK-FL25-C and PII-ST-V were coincubated with 2-OG for 30 min without preincubation and then the FRET ratio was determined. All signals were normalized to values from control experiments without 2-OG. All reactions were performed as triplicates; standard deviation is indicated by error bars. (C) ADP-induced dissociation of the NAGK-FL25-C + PII-ST-V complex, as determined by FRET analysis. Background subtracted and normalized to first 6 values. 0.1 mM ATP was present in the reaction mix. (D) End point measurements of ADP induced dissociation. Solid line: NAGK-FL25-C and PII-ST-V were incubated together for 10 min and then ADP was added. After 30 min of incubation the FRET ratio was determined. Dashed line: NAGK-FL25-C and PII-ST-V were coincubated with ADP for 30 min without preincubation and then the FRET ratio was determined. 0.1 mM ATP was present in the reaction mix, ADP concentrations were ranging from 0.25 to 8 mM. Inset: Magnification showing FRET signals from NAGK-FL25-C and PII-ST-V in the presence of 2 mM ATP and ADP concentrations ranging from 0.5 to 2 mM (dotted line) representing more physiological concentrations. All signals were normalized to values from control experiments without ADP. All reactions were performed as triplicates; standard deviation is indicated by error bars. (E) Dissociation of NAGK-FL25-C and PII-ST-V complex with 0.5 mM of malate, fumurate, succinate, oxalacetate, citrate, isocitrate and 2-OG. 0.075 mM ATP was present in all reactions.
