# Supplementary figures and images for: From PII Signaling to Metabolite Sensing: A Novel 2-Oxoglutarate Sensor That Details PII - NAGK Complex Formation
Source: PLoS One. 2013 Dec 12;8(12):e83181. doi: 10.1371/journal.pone.0083181 (PMC3861474; doi:10.1371/journal.pone.0083181)

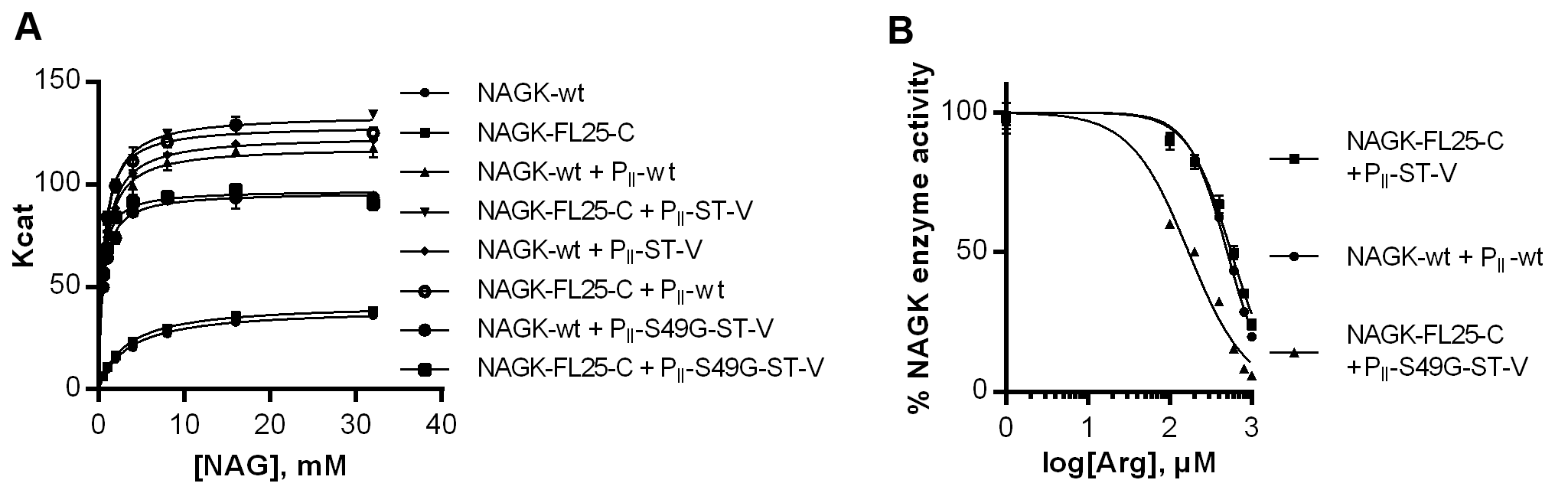

Supplement: Figure S1 — Determination of kinetic constants for NAGK-wt and NAGK‑FL25-C with and without various PII variants. (A) NAGK enzyme activity assays of the indicated PII - NAGK combinations were performed using increasing amounts of the substrate NAG. (B) NAGK enzyme activity assays of the indicated PII - NAGK combinations were performed with increasing concentrations of Arginine and 50 mM NAG. The mean of three replicas with error bars displaying the standard deviation is shown. The results are summarized in Tab. 1. (TIF) [file pone.0083181.s001.tif]

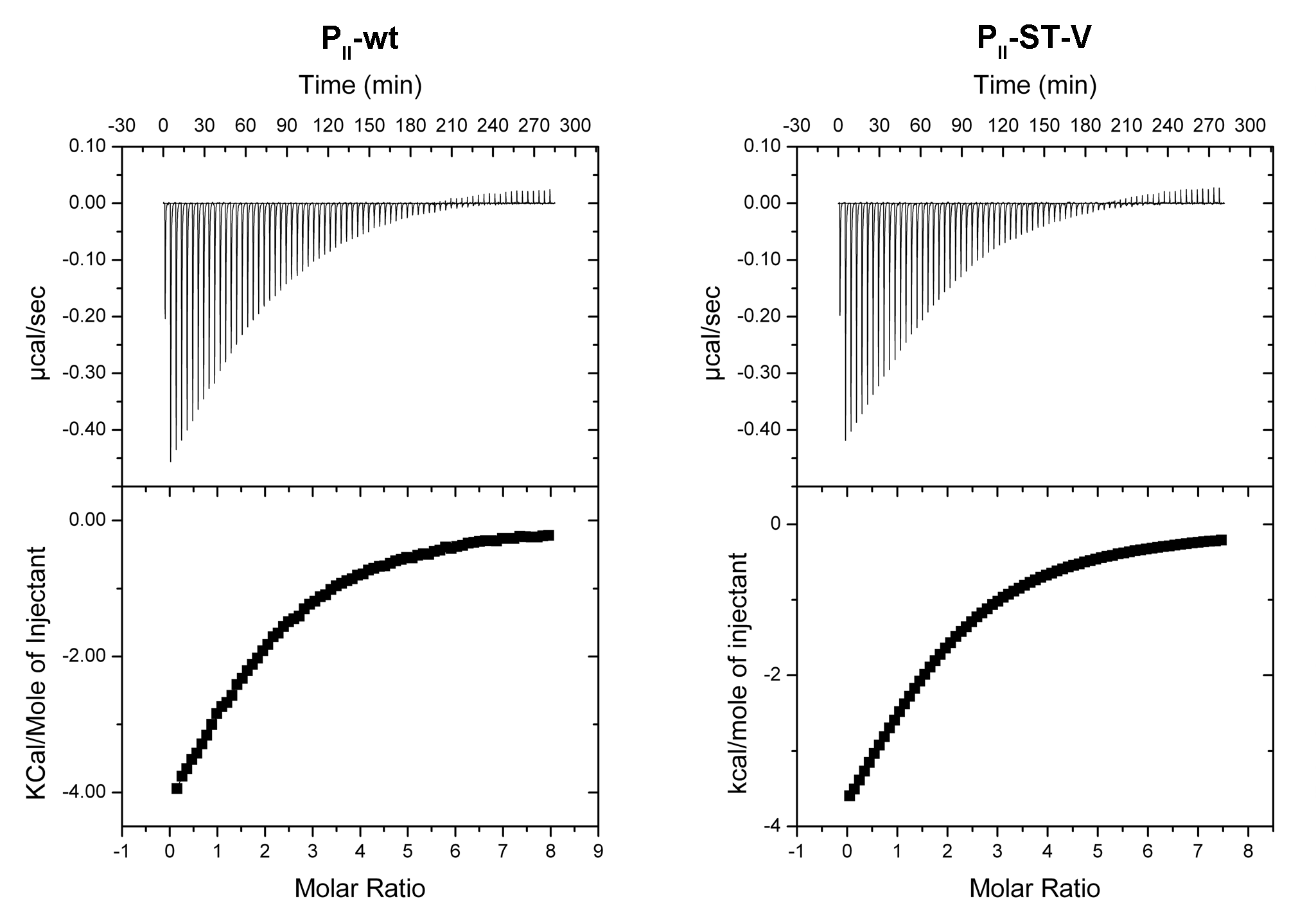

Supplement: Figure S2 — Two exemplary Isotherms for the determination of ATP binding properties of PII-wt and PII-ST‑V. The upper panel shows the raw data, the lower panel shows the calculated, blank subtracted, binding isotherms. The results are summarized in Table 2. (TIF) [file pone.0083181.s002.tif]

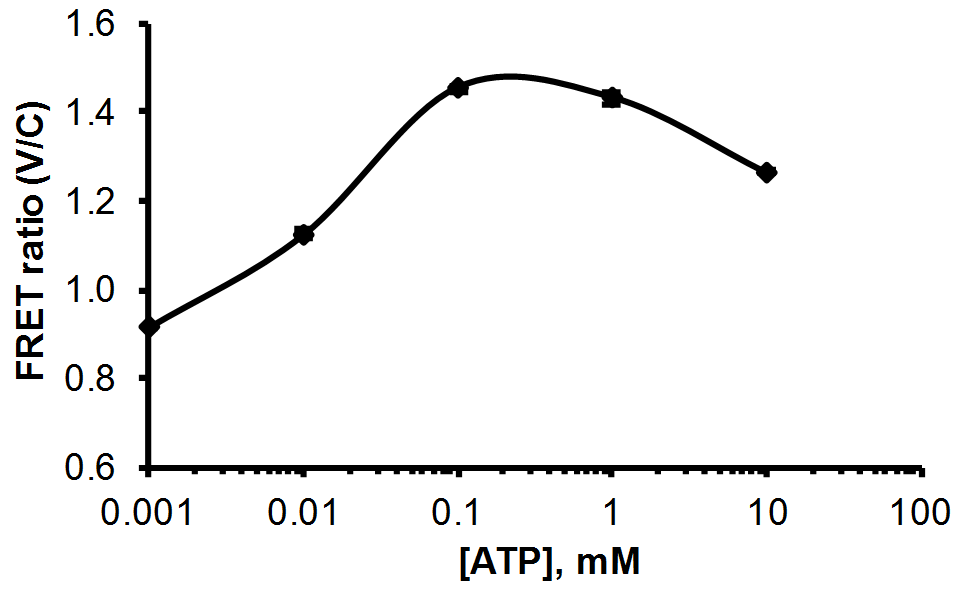

Supplement: Figure S3 — Effect of various ATP concentrations on FRET efficiency of NAGK‑FL25‑C with PII-ST‑V. The FRET ratio (525 nm / 475 nm emission) was measured after incubating NAGK-FL25-C and PII-ST-V together for 20 min at 37 °C in the presence of different ATP concentration. The mean of three replicas with error bars displaying the standard deviation is shown. (TIF) [file pone.0083181.s003.tif]

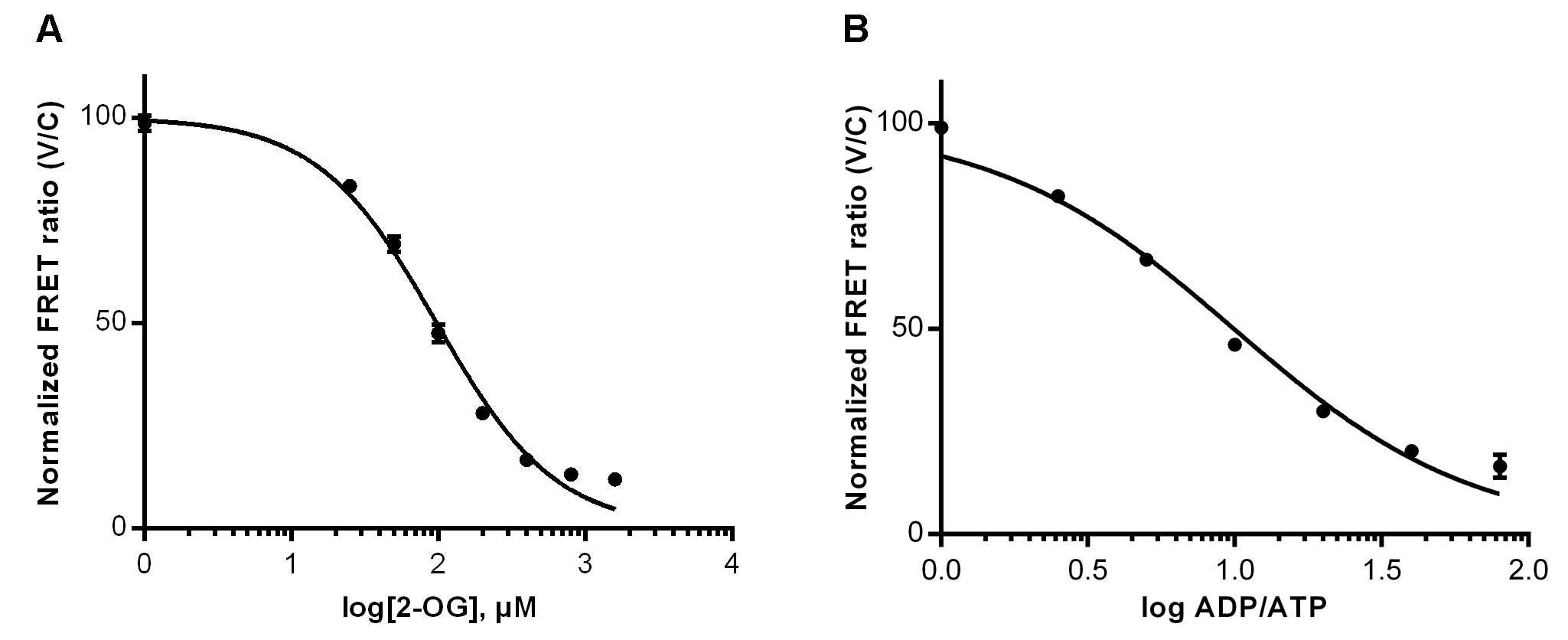

Supplement: Figure S4 — Determination of IC50 values for 2-OG and ADP on NAGK‑FL25‑C + PII-ST‑V. The relative FRET ratio (525 nm / 475 nm emission) was measured after incubating NAGK-FL25-C and PII-ST-V together for 30 min in the presence of different 2-OG concentration (A) or ADP/ATP ratios (B). All signals were normalized to values from control experiments without 2 OG or ADP. The mean of three replicas with error bars displaying the standard deviation is shown. Fitting and IC50 calculation was conducted using GaphPad Prism 6. (TIF) [file pone.0083181.s004.tif]

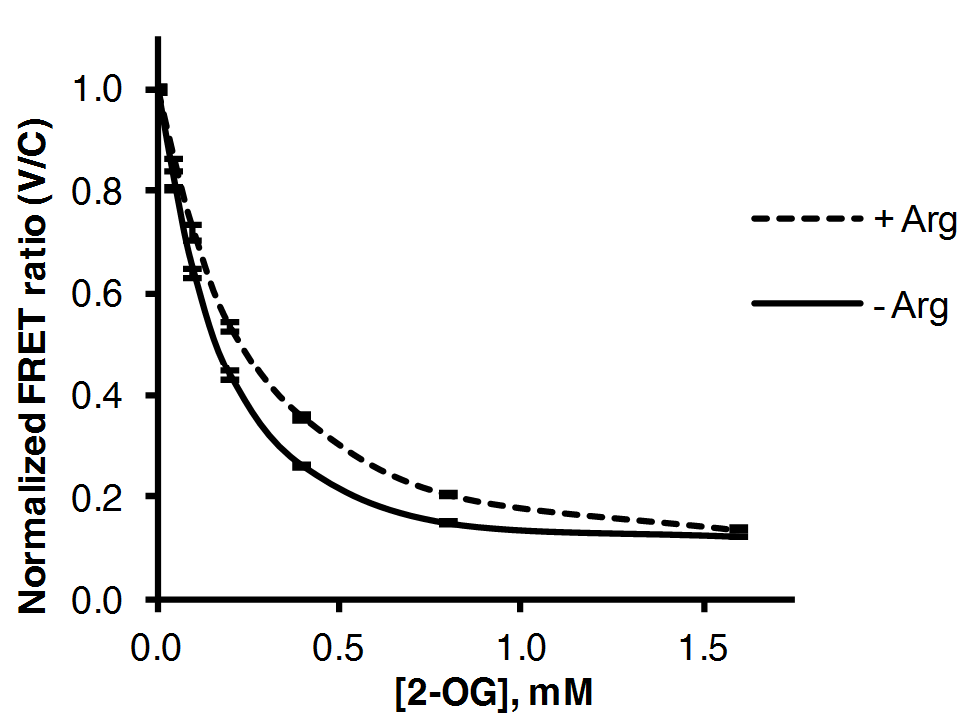

Supplement: Figure S5 — Influence of arginine on PII - NAGK complex sensitivity towards 2-OG. PII-ST-V and NAGK-FL25-C were incubated together for 40 min at 37 °C with different concentrations of 2-OG and with or without 1 mM arginine, before the FRET ratio was determined. All reactions were performed as triplicates; standard deviation is indicated by error bars. (TIF) [file pone.0083181.s005.tif]
